# Relationship between Health-Related Quality of Life and Physical Activity in Children with Hyperactivity

**DOI:** 10.3390/ijerph17082804

**Published:** 2020-04-18

**Authors:** Julio Gallego-Méndez, Jorge Perez-Gomez, José Ignacio Calzada-Rodríguez, Ángel Manuel Denche-Zamorano, María Mendoza-Muñoz, Jorge Carlos-Vivas, Miguel Ángel Garcia-Gordillo, Jose C. Adsuar

**Affiliations:** 1 Health, Economy, Motricity and Education Research Group (HEME), Faculty of Sport Sciences, University of Extremadura, 10003 Cáceres, Spain; julgalmen@gmail.com (J.G.-M.); jocalzada@alumnos.unex.es (J.I.C.-R.); andeza04@alumnos.unex.es (Á.M.D.-Z.); mamendozam@unex.es (M.M.-M.); jorge.carlosvivas@gmail.com (J.C.-V.); jadssal@unex.es (J.C.A.); 2 Facultad de Administración y Negocios, Universidad Autónoma de Chile, Sede Talca 3467987, Chile

**Keywords:** ADHD, HRQoL, physical activity, Spanish National Health Survey

## Abstract

The main purpose of this paper was to evaluate the relationship between health-related quality of life (HRQoL) and the frequency of physical activity in Spanish children aged 8 to 14 years with attention deficit hyperactivity disorder (ADHD). Sample selection was performed using the data obtained from the children’s questionnaire of the National Health Survey of Spain 2017 that is carried out with the children’s parents, and that had an initial size of 6106 participants. After the application of the inclusion and exclusion criteria, the sample size was reduced to 496 subjects. Results show significant differences between the different levels of physical activity frequency, as well as a positive correlation of the average between HRQoL and ADHD. In conclusion, the practice of physical activity may contribute to the improvement of HRQoL in children with ADHD, possibly achieving greater benefits at higher levels of physical activity practice.

## 1. Introduction

Hyperactivity refers to excessive movement and restlessness, usually involuntary, that does not respond to planned or constructive goals [1]. However, when this behavior is prolonged over time, regardless of the circumstances or conditions that generate it, and negatively interferes with the person’s personal, family, or social life, hyperactivity can be referred to as a pathological state [2]. Researches on this condition originally focused on the analysis of these symptoms as a consequence of trauma and brain damage [3]. Nevertheless, it was not until 1971 when Dawson and Satterfield proposed an alternative theory that justified the categorization of hyperactivity syndrome due to a deficit in the inhibitory control of the frontal cortex over limbic functions [4].

Currently, attention deficit hyperactivity disorder (ADHD) is defined as a neurobiological origin disorder that begins in childhood and is characterized by a high level of impulsive behavior, activity, and inattention, which is not appropriate to the age of development. There are different descriptions of ADHD symptoms that should be used for its diagnosis. For instance, the Diagnostic and Statistical Manual of Mental Disorders, Fifth Edition (DSM-V) distinguishes three dimensions [5], inattention, hyperactivity, and impulsivity, and establishes specific diagnostic criteria for each one of these dimensions, so subjects can be classified into subgroups based on the predominance of their symptoms. Also, there is another system developed by the World Health Organization (WHO) called the International Catalogue of Diseases (ICD), whose latest version is the ICD-10 [6]. It defines ADHD as “a group of disorders characterized by early-onset, the combination of hyperactive and poorly modulated behavior with a marked lack of attention and continuity in tasks and these problems occur in the most varied situations and persist over time”. It also distinguishes the dimensions of attention deficit, hyperactivity, and impulsivity. Different studies have indicated that the global prevalence of ADHD ranges between 4% and 8% in adults, and between 8% and 10% in children [7]. A meta-analysis by Polanczyk et al. [8] analyzed a total of 102 studies involving more than 170,000 under 19 years old young people and concluded that the worldwide rate of ADHD was close to 5%.

ADHD consequences differ according to different aspects such as degree of affectation; dominance of different dimensions; degree of control and treatment of the disorder; presence of comorbidities; and social, economic, and family characteristics of the participant’s environment [9,10]. Specifically, the main implications of children with ADHD are low academic performance; antisocial behavior; drug use and addictive behaviors; obesity; self-esteem problems; and limitations in executive function. Consequently, many children and adolescents with ADHD have difficulties regulating their behavior and adjusting to the expected norms and, therefore, have problems with their family, at school, and in their relationships with peers, which can lead to emotional and behavioral disorders [10,11,12].

Regarding the implications on life and the complexity of unifying the criteria to select assessments on people with ADHD, several authors have argued that health-related quality of life (HRQoL) is the most suitable indicator for analyzing the impact of this disorder on daily life and well-being [9,13,14,15,16]. HRQoL has been defined as a multidimensional construction that comprises several domains related to health and well-being, as it also refers to the subjective and objective impact of dysfunction associated with a disease or injury, medical treatment, and health status [17]. It incorporates the patient’s perception as a need in the evaluation of health outcomes regarding the physical, psychological, and social domains of health, seen as distinct areas that are influenced by a person’s experiences, beliefs, expectations, and skills [18]. The WHO established specific guidelines for HRQoL measurements: (1) to include the subjective component (i.e., people’s self-perception); (2) to be multidimensional (i.e., including information on physical, emotional, and social levels); (3) to consider positive and negative feelings; and (4) to record how feelings may vary over time since the vital stage or specific moment of an illness mark essential differences in the aspects to be valued [19]. Numerous studies have shown the relationship between physical activity and HRQoL in general populations [20,21,22], and also especially in children and adolescents [23,24].

Based on the above, it seems clear that higher levels of physical activity are related to greater levels of HRQoL and overall health benefits [22,25,26]. In fact, different studies have shown the benefits of regular physical activity on health and symptoms of people with ADHD [27,28,29]. Therefore, this study aimed to analyze the relationship between physical activity frequency and HRQoL in Spanish schoolchildren with ADHD aged between 8 and 14 years old, through the analysis of the data obtained by the National Health Survey of Spain 2017 (ENSE 2017) [30].

## 2. Materials and Methods 

### 2.1. Participants

**Figure 1:**
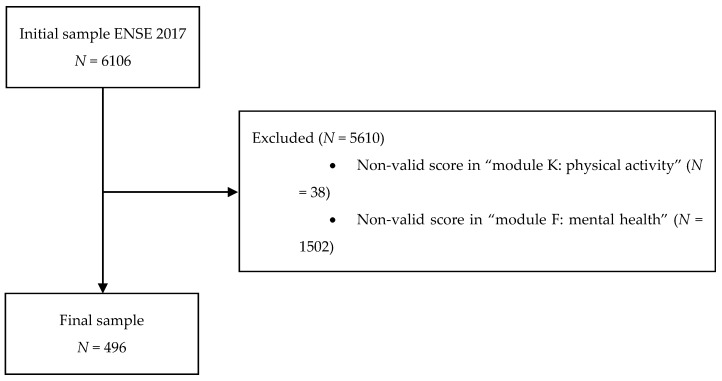
Sample selection process.

Sample selection was carried out using the data obtained from the ENSE 2017 questionnaire of children. This survey includes information collected from interviews conducted with the parents/guardians of children (8–14 years old), obtaining an initial size of 6106 participants. Initial contact with randomly selected households is made by sending a letter from the Ministry of Health, Consumer Affairs and Social Welfare requesting their collaboration, informing them that they have been selected for the survey, and reporting on the visit of an accredited interviewer. Additionally, the interview has two phases, which correspond with information related to demographic data and health issues respectively. In this way, data is collected through a computer-assisted personal interview (CAPI), which could be supplemented by a telephone interview when necessary and in exceptional cases. The information processing was carried out under the close supervision of the National Statistical Institute. After the application of inclusion and exclusion criteria, the sample size was reduced to 496 participants (Figure 1). The only inclusion criterion was having a determination of ADHD based on the score obtained in the “Hyperactivity” block of the Strengths and Difficulties Questionnaire (SDQ) (i.e., score ≥ 7). Moreover, the exclusion criteria were: (a) not having a valid score (values not between 1–4) for question 61 of “module K”, referring to physical activity habits, (b) not having a valid score (values not in the range of 1–3) on any of the “module F” questions related to mental health, and (c) not having a valid score (values not between 1–5) in any of the “module E” questions, referring to quality of life. The final sample included 496 Spanish children and adolescents (274 boys and 222 girls) aged between 8 and 14 years old (10.81 ± 2.04 years).

### 2.2. Evaluation Procedure and Instruments

This study only considered the following modules of ENSE 2017: “module E: Quality of Life”, “module F: Mental Health”, and “module K: Rest and Physical Activity” (only question 61 referring to physical activity frequency).

The “Block E: Quality of Life” assesses HRQoL of the child population from the child’s perspective in terms of physical, mental, and social well-being, and allows for the identification of the populations at risk in terms of their subjective health. To do this, the parental version of the Kidscreen-10 instrument adapted for Eurobarometer was used, removing specifically question 7 from the original version: Have the parents of the child or adolescent treated him/her fairly? [31,32,33]. Thus, the questions were asked of an indirect informant (parents/guardians) who had to answer what they thought the child would answer. These questions refer to the frequency in which children have gone through the different situations over the last week, based on the time reference. Possible answers are “nothing” (1), “a little” (2), “moderately” (3), “very much” (4) and “a lot” (5). Both the original 10-item version (α de Cronbach = 0.82) and the modified 9-item version (α de Cronbach = 0.75) of Kidscreen-10 has shown good psychometric properties in European children and adolescents. Also, the Spanish version has acceptable reliability and validity (α de Cronbach > 0.70) [32,34,35,36].

The “Block F: Mental Health” questions aims to assess the prevalence of risk of poor mental health in children using the parents’ version of the SDQ [37]. It is a brief behavioral assessment questionnaire for children and adolescents aged from 3 to 16 years, which was designed to be used as a screening instrument for the main psychopathologies in children and adolescents, according to the diagnostic categories established in the international classification systems [38]. The tool consists of emotional symptoms (5 items), behavior problems (5 items), hyperactivity (5 items), peer problems (5 items), and prosocial behavior (5 items). The child’s parents must answer each question based on the last six months. Possible answers are “not true” (1), “somewhat true” (2), and “absolutely true” (3).

Since ENSE 2017 does not include a specific item that directly refers to the diagnosis of ADHD, the score corresponding to the “Hyperactivity” block of the SDQ was used in the present study to assess the presence of ADHD. Previous studies have supported the use of this SDQ dimension to evaluate the presence of ADHD in children [39,40,41,42,43]. This "hyperactivity" subscale consists of five items (three items about the "hyperactivity/impulsivity" dimension and two about the “inattention” dimension), according to the diagnostic criteria established in both the ICD-10 [6] and DSM-V [5]. Most authors agree in pointing out that a score ≥7 in this block is the cut-off point for the presence of ADHD in children, obtaining acceptable sensitivity and specificity values (both ≥ 74%) in similar samples to the one in this study [44,45,46,47,48].

The ENSE 2017 of children only includes one question regarding physical activity frequency (i.e., question 61, corresponding to “Block K: Rest and Physical Activity”). Therefore, the aim was to quantify physical activity volume and assess whether the population complies with the WHO physical activity recommendations. For this purpose, the short version of the adapted International Physical Activity Questionnaire [49] was used, which is limited to a single question referring to the practice of physical activity in free time in the children’s ENSE 2017. The question has four possible answers [50]: (1) “no exercise” (free-time mainly occupied by sedentary activities such as reading, watching television, going to the cinema…); (2) “occasional physical activity or sport” (walking or cycling, gentle gymnastics, recreational activities that require a slight effort…); (3) “physical activity several times a month” (sports, gymnastics, running, swimming, cycling, team games…); and (4) “sports or physical training several times a week”.

### 2.3. Variables Used in the Analysis

MAPPING CHU9D: This is a derived variable that estimates HRQoL from the transformation of results obtained from the items corresponding to “Block E: Quality of Life” (E14_1, E14_2, E14_3, E14_4, E14_8, and E14_9) of the Kidscreen-10 Index modified proxy for the Eurobarometer [31,32,33], obtained using the following equation [51]:

MAPPING CHU9D = 0.222655 + 0.037867 * E14_1 + 0.023085 * E14_2 + 0.037192 * E14_3 + 0.021284 * E14 _4 + 0.024877 * E14_8 + 0.022256 * E14_9

The scores for this variable are collected on a scale of 0 to 1, where 0 refers to death, and 1 refers to the highest possible level of HRQoL. CHU9D is a preference-based instrument suitable for application in economic evaluations of health interventions [52,53].

KS9: This is a derived variable that assesses HRQoL of children and adolescents aged from 8 to 14 years old. Computations are carried out from the outcomes obtained in the "Block E: Quality of Life" using the Kidscreen-10 Index modified proxy for the Eurobarometer [31,32,33]. Due to the different number of items compared to the original Kidscreen-10 questionnaire (9 items as opposed to the original 10), it was necessary to rename the variables corresponding to the scores on each of the items to adequately perform the calculation of a summary measure of HRQoL. The syntax recommended by the European Kidscreen Group was applied to calculate HRQoL from only nine items [54]. This same syntax was used previously in other research to estimate the variable that summarizes HRQoL based on the nine items of the modified Kidscreen-10 in European children and adolescents [36]. The value of this HRQoL variable can range from 1 to 5, with higher values representing higher HRQoL levels.

PA (K61 in ENSE 2017): This tool collects information on the physical activity performed by the child in his/her free time, according to the frequency with which he/she performs it. This value is obtained from the score obtained in question 61 of “Block K: Rest and Physical Activity”. The possible values of this variable are: 1 “the child does not exercise”, 2 “the child does some occasional physical activity or sport”, 3 “the child does physical activity several times a month”, or 4 “the child does sports or physical training several times a week”.

### 2.4. Statistical Analysis

Statistical analyses were performed using IBM SPSS Statistics 20 software (IBM, Armonk, NY, USA). Descriptive statistics were computed for the characterization of the sample, both for the total sample and for different subdivisions made according to sex (girls and boys) and by age subgroups (8–12 and 13–14 years old). Data are presented as mean, standard deviation (SD), median, and interquartile range (IR) for age, PA, KS9, and MAPPING CHU9D.

Normality tests were carried out applying the Kolmogorov–Smirnov test to check the data distribution along with the different variables. After using these tests, a *p* value < 0.0001 was obtained for *p* = 0.05 in all the analyses. For the dataset with different variables, it was necessary to apply non-parametric tests for carrying out the subsequent statistical analyses. To evaluate possible differences between subgroups for each variable, the Mann–Whitney U non-parametric test was used for two independent samples, both for comparisons between age subgroups and between sexes, as well as for the comparison between age subgroups within each sex.

Between-subgroup comparison in HRQoL (KS9 and MAPPING CHU9D, respectively) was also carried out according to the frequency of physical activity measured in PA. Also, to evaluate the existence of significant differences between HRQoL levels according to PA, the Kruskal–Wallis non-parametric test was performed for the KS9 and MAPPING CHU9D variables using the different levels of the PA variable as factors. For the post-hoc analysis, the Mann–Whitney U test was used for the pairwise comparisons, with the corresponding Bonferroni’s correction to counteract the problem of multiple comparisons, establishing *p* < 0.017 [55]. Likewise, Mann–Whitney’s non-parametric U test for two independent samples for the MAPPING CHU9D and KS9 was applied to assess possible differences in HRQoL between children and adolescents with ADHD who engage in physical activity (scores 2–4 in K61) and who do not (score 1 in K61).

Finally, a bivariate correlation was made to confirm the level of association between HRQoL and PA in subjects with ADHD, applying the Spearman’s non-parametric correlation coefficient, among PA, KS9, and MAPPING CHU9D variables. Thus, the respective Spearman’s correlation coefficients were calculated between PA and every item corresponding to Kidscreen-10 Index modified proxy questionnaire for the Eurobarometer, together with the significance level of every value.

## 3. Results

Table 1 shows the descriptive statistics for the sample as a whole (*n* = 496); by subgroups according to sex: boys (*n* = 274) and girls (*n* = 222); by subgroups according to age: 8–12 years (*n* = 366) and 13–14 years (*n* = 130); and by age subgroups within each subgroup depending on sex: 8–12 years boys (*n* = 200), 13–14 years boys (*n* = 74), 8–12 years girls (*n* = 166), and 13–14 years girls (*n* = 56). The mean age of the sample was 10.81 (±2.04) years with a median of 11 ± 4 years.

Regarding PA, mean and median values close to 3 were obtained for both total sample and subgroups of boys and girls separately. This value corresponds to a frequency of physical activity per month and constitutes the second-highest value on the frequency measurement scale used in the questionnaire (values 0, 1, 2, 3, and 4). Significant differences were observed (*p* < 0.05) in the frequency of physical activity between sexes, being slightly lower in girls. No significant differences were found among the rest of the subgroups for PA.

Regarding the HRQoL measured using the summary variable KS9, an average of 4.52 (±0.41) and a median of 456 (0.61) were observed. Five is the maximum possible value for this variable, which corresponds to the highest HRQoL level and 0 the minimum potential value. There were significant differences in the values of this variable between age subgroups in the total sample (*p* = 0.034) and between age subgroups within children (*p* = 0.027) although with weak significant values. No significant differences were seen between the rest of the subgroups for the variable KS9.

The results of the descriptive statistical analysis for the variable MAPPING CHU9D showed mean 0.78 (±0.07) and median 0.78 (0.08). This variable, as indicated above, estimates the HRQoL by assigning values between 0 (lowest possible HRQoL) and 1 (highest possible HRQoL). As in the frequency of physical activity, significant differences were found in the value of this variable between boys and girls (*p* = 0.014) with girls having a better HRQoL. In the rest of the subgroups, no significant differences were observed.

Table 2 and Table 3 show between-subgroup differences in HRQoL level results (KS9 and MAPPING CHU9D, respectively) according to physical activity frequency measured by the PA variable.

There is a continuous and progressive increase in the mean values and the median HRQoL as the frequency of physical activity increases, measured by both KS9 and MAPPING CHU9D. Also, to evaluate the existence of significant differences between HRQoL levels according to the frequency of physical activity, the Kruskal–Wallis’ non-parametric test was performed for the KS9 and MAPPING CHU9D variables using the different levels of the PA variable as factors. In both cases, *p* < 0.005.

Since, in both cases, the result of the Kruskal–Wallis test indicated the existence of significant differences in at least one of the medians, post-hoc analyses were performed using the Mann–Whitney U test for the KS9 and MAPPING CHU9D variables to detect among which specific subgroups of physical activity frequency these significant differences occur. In these analyses, Bonferroni’s corrections were applied to counteract the problem of multiple comparisons by obtaining *p* < 0.17 [55].

The results of these post-hoc analyses show that the significant differences in HRQoL are mainly among subgroups that do not engage in any physical activity compared to those that do so several times a week. Although, as described above, by comparing average HRQoL values between subgroups, it can be observed how the quality of life values increase as the frequency of physical activity increases for all subgroups. Therefore, to complement the information obtained with the previous tests, we proposed the comparison between the HRQoL levels of students with ADHD who do not engage in physical activity (value 1 in PA) and those who do (values 2, 3, and 4 in PA) using the variables KS9 and MAPPING CHU9D.

This analysis was carried out using the non-parametric Mann–Whitney U test, and the results are shown in Table 4. There were significant differences between these two groups, both for the KS9 variable (*p* = 0.003) and the MAPPING CHU9D variable (*p* = 0.002). These results confirm that there are differences in the HRQoL of students with ADHD who do not engage in any physical activity and those who do.

Once the existence of significant differences in HRQoL values between groups according to the frequency of physical activity was verified, it was necessary to evaluate the type of relationship between PA and HRQoL levels (KS9 and MAPPING CHU9D).

For this purpose, the non-parametric bivariate correlation was performed using Spearman’s correlation coefficient between these variables: The results are shown in Table 5. For interpreting the data from the table on the degree of relationship according to the Spearman’s correlation coefficient, the Mondragón–Barrera method was used, based on Sampieri et al., so that values between 0.11 and 0.50 were considered to be an average positive correlation [56,57].

Spearman correlation coefficient was calculated both generally for the sample as a whole and independently for the boys and girls in the sample (Table 5). Also, the calculation was made for the subgroups of age within each sex. A significant correlation was obtained for both the variable KS9 (R = 0.151) and MAPPING CHU9D (R = 0.146) concerning PA for total sample analysis. Both values showed a direct positive correlation between the variables corresponding to HRQoL and physical activity and were within the values indicated for considering the existence of an average positive relationship [56,57]. Similarly, in the calculation of the respective correlation coefficients of the boys in the sample, significant correlations were obtained with a value of R=0.148 for the association between KS9 and PA, and value of R=0.178 for the relationship between MAPPING CHU9D and PA. Also, in the case of girls, values were obtained that can be considered comparable to the comparisons of boys (R = 0.177 and R = 0.143, respectively).

There was a direct correlation between PA and HRQoL for both the total sample and the subsample of boys. In the case of girls, a direct correlation between PA and HRQoL was found in those aged 13-14, but this correlation was not found in girls aged 8–12, probably because of the smaller number of participants in these subgroups.

Table 6 shows the values of the respective Spearman’s [58] correlation coefficients calculated between the PA variable and each of the items corresponding to the Kidscreen-10 Index modified proxy questionnaire for the Eurobarometer used to measure HRQoL, together with the significance level of each of the values.

## 4. Discussion

One of the findings of this study is that boys, aged 8-14 years old, with ADHD engage in physical activity more frequently than girls (Table 1). These results are in line with previous studies in the general population of children, in which boys were more physically active than girls [59,60,61]. On the other hand, in organized sports activities, boys seem to spend more time in physical activity of moderate to vigorous intensity than girls [62].

Another of the study’s findings is that children with ADHD have a decrease in HRQoL from primary to secondary school education (Table 1). This result is in line with other previous studies that found that HRQoL decreased as age increased in children [63]. Traditionally, the transition from primary to secondary education is associated with a drop in sports-related activities [64,65,66]; we only found a significant difference for boys not in girls in terms of a decrease in activity with increased age. Speculating on the possible reasons for the decline in HRQoL at the beginning of high school, we think this could be due to: (1) adaptation problems to the higher level of education, (2) changes in personal interest during adolescence, such as going out socially on the weekends instead of practicing physical activity or sports competitions.

The main outcome of this study is that a greater frequency of physical activity may contribute to a higher HRQoL in children with ADHD (Table 5). According to the Spanish National Health Survey conducted in 2017 (Table 2), there is a significant relationship, based on an average positive correlation, between physical activity frequency and the HRQoL in children between 8 and 14 years old with ADHD. On the other hand, it has been found that sedentary children with ADHD show worse HRQoL than those who are physically more active (Table 4). Specifically, we found a significant difference between sedentary children and those who engage in physical activity, and also between sedentary children and those who practice physical activity several times a month (Table 3). Our results are concordant with previous studies that showed the positive effects of physical activity on health and quality of life of children and adolescents with [27,28,67,68,69] or without ADHD [70,71]. Additionally, the results obtained regarding the relationship between the frequency of physical activity and HRQoL in the group of children with ADHD were similar to those of the rest of children without ADHD in the National Health Survey conducted in 2017. Therefore, physical activity relates to improved quality of life for both ADHD and non-ADHD populations, but physical activity may benefit ADHD symptoms.

HRQoL can be defined as *the physical, mental and social effects of the illness on daily life* [72], therefore, the improvements in HRQoL in both children and adolescents without and with ADHD could be explained by the improvements produced by physical activity in the domains of physical condition, mental health, and social well-being. One meta-analyses whose objective was to study the relationship between physical activity and fitness concluded that *the available evidence supports a link between muscular fitness and physical activity in children and adolescents* [24]. A review of research about physical activity and mental health in children and adolescents concluded that *evidence shows small but consistent associations between sedentary screen time and poorer mental health* [73] and another review concluded that *physical activity interventions can improve adolescents’ mental health* [74]. Finally, a review of the literature focused on social benefits of participation in sports for children and adolescents *suggested that there were many different social health benefits reported, with the most commonly being improved self-esteem and social interaction* [75].

Although the specific symptoms of children and adolescents with ADHD have not been assessed in this study, here are some of the reasons that may explain why those who engage in physical activity more often may have a better HRQoL: (1) beneficial effects of physical activity on some symptoms of ADHD, (2) decrease in comorbidities that usually occur in children and adolescents with ADHD due to increased levels of physical activity, (3) improvement in inhibitory control and executive function, (4) improvement in cognitive function, (5) improvement in social competence, and (6) improvement in motor control. Each of these points will be developed below.

1. Beneficial effects of physical activity on some ADHD symptoms. Some studies have described the effects of physical activity on ADHD symptoms, highlighting that the mechanisms of action are similar to those of commonly prescribed ADHD medications and show synergistic effects as complementary therapy [76,77,78].

2. Decreased comorbidities. Some authors have suggested that regular practice of physical activity in children and adolescents with ADHD represents a protective factor against the frequent comorbidities of ADHD such as anxiety, depression, or obesity [79,80], which directly affect the quality of life of these people, as shown by the results of this study.

3. Improved inhibitory control and executive function. Smith et al. [81] reported benefits in motor, cognitive, social, and behavioral functioning related to physical activity in children with ADHD. They noted significant changes in inhibitory control and executive function, as these two aspects are mainly affected in people with this disorder and largely induce lower levels of quality of life [11,12,82].

4. Improvement of cognitive function. Significant improvements have been shown in cognitive function, behavior, and academic performance of students with ADHD who were engaged in high levels of physical activity [83]. All these processes are closely linked to the child’s quality of life, since they have a high impact on acceptance and recognition in the immediate environment from which the child builds his or her self-concept and self-esteem [10,80,82]. On the other hand, the modified Kidscreen-10 proxy questionnaire [31,33,34], used to measure quality of life in the ENSE 2017 standard, refers in questions eight and nine to cognitive function and academic performance at school, finding a statistically significant direct correlation between levels of physical activity and feeling good at school, however, no correlation was found in the case of question nine regarding being able to pay attention (Table 6).

5. Improvement in social competence. One of the most relevant markers related to HRQoL in children and adolescents with ADHD is their social behavior, linked to their social skills when relating mainly to their peers [12,13,27]. The modified Kidscreen-10 proxy questionnaire makes direct reference to the child’s social relationships. More specifically, question number seven asks if the child has had fun in the last week with his or her friends, and question number four, if he or she has felt alone recently. A statistically significant direct correlation was found between the frequency of physical activity and having fun with friends (Table 6). Verret et al. [84] reported considerable improvements in children’s social skills and peer relationships when they follow a regular physical activity program. Kang et al. [85] observed similar results in improving social skills and social competence in children with ADHD who significantly increased their level of physical activity and sports. Other studies have shown similar improvements in behavior and social competence [27,68]. Therefore, the literature supports the results found in this research to the extent that improvement in social skills due to physical activity practice has an impact on higher scores on the modified Kidscreen-10 proxy questionnaire, and therefore on the HRQoL measures used in the study.

6. Improvement in motor control. Other aspects that improve significantly with regular practice of physical activity in people with ADHD are related to motor control, locomotor skills, and physical fitness of students [27,67,79,84,86]. Questions number one and two of the modified Kidscreen-10 proxy questionnaire refer to whether the child has felt good, fit, and full of energy.

Finally, after analyzing the results of this study related to HRQoL in ENSE 2017 and comparing them with others reported in the scientific literature, it can be stated that higher levels of physical activity practice are related to higher levels in HRQoL of children with ADHD. However, this study only analyses the existence of a relationship between both measures and does not explain the mechanisms for the increase in HRQoL levels. A limitation of the study is that specific symptoms of ADHD were not measured and therefore strong conclusions cannot be made to suggest physical activity reduces symptoms of ADHD.

Therefore, future studies should focus on aspects that could influence HRQoL, how the regular practice of physical activity can improve HRQoL, and learning more about the mechanisms by which physical activity could improve HRQoL in this specific population.

## 5. Conclusions

1. Boys, aged 8–14 years old with ADHD may be more likely to engage in physical activity than girls of the same age with ADHD.
2. There may be a decline in HRQoL in the transition from primary to secondary school education in children with ADHD.
3. The practice of physical activity may contribute to the improvement of HRQoL in children with ADHD; it may be possible to achieve greater benefits at higher levels of physical activity practice.
4. Increased frequency of physical activity in children aged 8–14 with ADHD could be related to feeling good and fit; feeling full of energy, having more fun with friends, and doing better in school.

## Figures and Tables

**Table 1:** Sample characteristics.

Variables	TOTAL				BOYS				GIRLS				
**AGE**	8–12 (*N* = 366)	13–14 (*N* = 130)	8–14 (*N* = 496)	***p***	8–12 (*N* = 200)	13–14 (*N* = 74)	8–14 (*N* = 274)	***p*** *	8–12 (*N* = 166)	13–14 (*N* = 56)	8–14 (*N* = 222)	p *	p **
Median (IR)	10 (2)	14 (1)	11 (4)	n/a	10 (2)	14 (1)	11 (4)	n/a	10 (2)	14 (1)	11 (4)	n/a	0.763
Mean (SD)	9.83 (1.38)	13.56 (0.49)	10.81 (2.04)		9.82 (1.35)	13.57 (0.49)	10.83 (2.04)		9.84 (1.41)	13.55 (0.50)	10.77 (2.04)		
**PA**													
Median (IR)	3 (2)	3 (2)	3 (2)	0.113	3 (2)	3 (1)	3 (2)	0.068	3 (2)	3 (2)	3 (2)	0.768	**0.005**
Mean (SD)	2.83 (1.03)	2.98 (1.02)	2.87 (1.03)		2.93 (0.99)	3.16 (0.95)	2.99 (0.98)		2.70 (1.07)	2.75 (1.08)	2.72 (1.15)		
**KS9**													
Median (IR)	4.69 (0.56)	4.44 (0.67)	4.56 (0.61)	**0.034**	4.61 (0.56)	4.44 (0.78)	4.56 (0.67)	**0.027**	4.67 (0.56)	4.56 (0.56)	4.56 (0.56)	0.501	0.081
Mean (SD)	4.54 (0.39)	4.44 (0.44)	4.52 (0.41)		4.52 (0.41)	4.37 (0.47)	4.48 (0.43)		4.57 (0.36)	4.53 (0.39)	4.56 (0.36)		
**MAPPING CHU9D**													
Median (IR)	0.78 (0.08)	0.77 (0.09)	0.78 (0.08)	0.313	0.77 (0.10)	0.76 (0.10)	0.77 (0.10)	0.903	0.80 (0.07)	0.78 (0.09)	0.80 (0.08)	0.185	**0.014**
Mean (SD)	0.78 (0.06)	0.77 (0.06)	0.78 (0.06)		0.77 (0.07)	0.77 (0.06)	0.77 (0.06)		0.79 (0.06)	0.77 (0.05)	0.78 (0.06)		

* The difference between boys and girls aged 8–12 and 13–14 years is noted, performing a non-parametric test for independent variables obtaining the *p* of the Mann–Whitney U test for *p* = 0.05. ** The difference between boys and girls is indicated, performing a non-parametric test for independent variables obtaining the *p* of Mann–Whitney’s U test for *p* = 0.05; n/a = not applicable. AGE: age measured in years. PA: physical activity frequency (values 1–4). KS9: derived variable for the HRQoL (values 1–5). MAPPING CHU9D: derived variable that estimates the HRQoL (values 0–1).

**Table 2:** Comparison of HRQoL, calculated by KS9, according to PA.

PA		KS9	PA	MEANS DIF	MEDIANS DIF	*p* *	p **
**1**	Median (IR) Mean (SD)	4.37 (0.45) 4.44 (0.56)	2	−0.11	−0.11	**0.005**	0.092
			3	−0.15	−0.11		0.023
			4	−0.21	−0.22		**<0.001**
**2**	Median (IR) Mean (SD)	4.49 (0.46) 4.56 (0.78)	1	0.11	0.11	**0.005**	0.092
			3	−0.04	0.00		0.824
			4	−0.09	−0.11		0.159
**3**	Median (IR) Mean (SD)	4.53 (0.36) 4.56 (0.45)	1	0.15	0.11	**0.005**	**0.023**
			2	0.04	0.00		0.824
			4	−0.05	−0.11		0.052
**4**	Median (IR) Mean (SD)	4.58 (0.40) 4.67 (0.50)	1	0.21	0.22	**0.005**	**<0.001**
			2	0.09	0.11		0.159
			3	0.05	0.11		0.052

* Kruskal–Wallis global for a *p* = 0.05, using as an answer, the HRQoL according to KS9, and as a factor, the level of physical activity PA: 1 (The child does not exercise.), 2 (The child does some occasional physical activity or sport), 3 (The child does physical activity several times a month), or 4 (The child does sports or physical training several times a week). ** Mann–Whitney post hoc U analysis with Bonferroni correction factor having *p* = 0.017. KS9: derived variable for the HRQL (values 1–5). PA: frequency of physical activity (values 1–4). MEAN DIF: difference between the average values of KS9 for each level of PA. MEDIANS DIF: difference between the median values of KS9 for each PA level.

**Table 3:** Comparison of HRQoL, calculated by MAPPING CHU9D, according to PA.

PA		MAPPING CHU9D	PA	MEAN DIF	MEDIANS DIF	p*	p**
**1**	Median (IR) Mean (SD)	0.76 (0.07) 0.75 (0.09)	2	−0.01	−0.02	**0.005**	0.206
			3	−0.03	−0.05		**0.002**
			4	−0.03	−0.05		**0.001**
**2**	Median (IR) Mean (SD)	0.77 (0.07) 0.77 (0.11)	1	0.01	0.02	**0.005**	0.206
			3	−0.01	−0.02		0.206
			4	−0.02	−0.03		0.083
**3**	Median (IR) Mean (SD)	0.78 (0.06) 0.80 (0.08)	1	0.03	0.05	**0.005**	**0.002**
			2	0.01	0.02		0.206
			4	−0.01	−0.01		0.537
**4**	Median (IR) Mean (SD)	0.78 (0.06) 0.80 (0.09)	1	0.03	0.05	**0.005**	**0.001**
			2	0.02	0.03		0.083
			3	0.01	0.01		0.537

* Kruskal–Wallis global for a *p* = 0.05, using as an answer, the HRQoL according to KS9, and as a factor, the level of physical activity PA: 1 (The child does not exercise.), 2 (The child does some occasional physical activity or sport), 3 (The child does physical activity several times a month), or 4 (The child does sports or physical training several times a week). ** Mann–Whitney post hoc U analysis with Bonferroni correction factor having *p* = 0.017. MAPPING CHU9D: derived variable that estimates the HRQoL (values 0–1). PA: frequency of physical activity (values 1–4). MEAN DIF: difference between the average values of MAPPING CHU9D for each PA level. MEDIANS DIF: difference between the medians of MAPPING CHU9D for each PA level.

**Table 4:** Comparison of HRQoL values between children and adolescents with attention deficit hyperactivity disorder who do not engage in physical activity and those who do using the KS9 and MAPPING CHU9D variables.

Variables	Do not Engage in Physical Activity (*N* = 72)	Do Engage in Physical Activity (*N* = 424)	p *
**KS9**			
Median (IR)	4.44 (0.56)	4.61 (0.55)	**0.003**
Mean (SD)	4.37 (0.45)	4.54 (0.39)	
**MAPPING CHU9D**			
Median (IR)	0.75 (0.10)	0.78 (0.08)	**0.002**
Mean (SD)	0.76 (0.07)	0.78 (0.06)	

* Mann–Whitney U for two independent samples for *p* = 0.05. **KS9:** derived variable for the HRQoL (values 1–5). **MAPPING CHU9D**: derived variable that estimates the HRQoL (values 0–1). **Do not engage in physical activity:** value 1 in the PA variable (frequency of physical activity). **Do engage in physical activity:** values 2–4 in PA variable (frequency of physical activity).

**Table 5:** Pearson’s bivariate correlation between PA and HRQoL (KS9 and MAPPING CHU9D).

Variable	PA
**KS9**	*rho*
TOTAL	0.151 **
BOYS	0.148 *
8–12	0.143 *
13–14	0.214 *
GIRLS	0.177 **
8–12	0.133
13–14	0.291 *
**MAPPING CHU9D**	*rho*
TOTAL	0.146 **
BOYS	0.178 **
8–12	0.197 **
13–14	0.109 *
GIRLS	0.143 *
8–12	0.062
13–14	0.441 **

The correlation is significant at the 0.01 level (bilateral). * Correlation is significant at the 0.05 level (bilateral) KS9: derived variable for the HRQoL (values 1–5). ** Correlation is significant at the 0.01 level. MAPPING CHU9D: derived variable that estimates the HRQoL (values 0–1). PA: frequency of physical activity (values 1–4).

**Table 6:** Correlation table between the PA variable (physical activity frequency) and each of the items of the Kidscreen-10 Index modified for the Eurobarometer.

Questions	Spearman’s Correlation Coefficient	*p*
Has your child felt fit and well?	0.133 *	**0.003**
Has your child felt full of energy?	0.134 *	**0.003**
Has your child felt sad?	−0.041	0.528
Has your child felt lonely?	−0.045	0.329
Has your child had enough time for him/herself?	0.019	0.667
Has your child been able to do the things that he/she wants to do in his/her free time?	0.064	0.152
Has your child had fun with his/her friends?	0.155 **	**0.001**
Has your child got on well at school?	0.091 *	0.044
Has your child been able to pay attention?	0.072	0.107

* The correlation is significant at the level *p* < 0.05 (bilateral). ** The correlation is significant after applying the Bonferroni’s correction factor having a *p* = 0.0056. Kidscreen-10 Index questionnaire modified for the Eurobarometer from 1 to 9.
